# Klotho ameliorates oxidized low density lipoprotein (ox-LDL)-induced oxidative stress via regulating LOX-1 and PI3K/Akt/eNOS pathways

**DOI:** 10.1186/s12944-017-0447-0

**Published:** 2017-04-13

**Authors:** Yansheng Yao, Yanbing Wang, Yibo Zhang, Chang Liu

**Affiliations:** 1 Jinzhou Medical University, Jinzhou, Liaoning China; 2 Department of Pathogenic Biology, Jinzhou Medical University, Jinzhou, Liaoning China; 3 Department of Endocrinology, First Affiliated Hospital of Jinzhou Medical University, Jinzhou, Liaoning China

**Keywords:** Klotho, Oxidized low density lipoprotein, Oxidative stress, Lectin-like ox-LDL receptor, Human umbilical vein endothelial cells

## Abstract

**Background:**

Atherosclerosis is a common cardiovascular disease that causes myocardial infarction, heart failure, and stroke. Increased oxidized low density lipoprotein (ox-LDL) in the sub-endothelium is the characteristic origin of atherogenesis. Klotho, an anti-aging protein, has been reported to protect against atherosclerosis and ameliorate endothelial dysfunction in vivo. The aim of this study is to investigatethe anti-oxidative activity of Klothoin ox-LDL-treated human umbilical vein endothelial cells (HUVECs).

**Methods:**

After pre-treatment with 200 pMKlotho for 1 h, HUVECs were stimulated with 50 μg/ml ox-LDL for 24 h. Reactive oxygen species (ROS) and superoxide dismutase (SOD) levels were analyzed in the cells. Nitric oxide (NO) concertation was measured in the medium supernatant. Related proteins or genes were detected with Western blot or real time PCR, respectively, in the cell lysates.

**Results:**

Initially, oxidative damage in HUVECs was established by adding 50 μg/mL ox-LDL, which resulted in decreased cellular viability, SOD/Cu/Zn-SOD and endothelial NO synthase (eNOS) expression and NO production, as well as increased malondialdehyde (MDA) levels, ROS production, inducible NO synthase (iNOS), phosphatidyl inositol-3 kinase (PI3K), protein kinase B (Akt), gp91 phox, and lectin-like ox-LDL receptor (LOX-1) expression in HUVECs. Pre-incubation with recombinant Klotho (200 pM) significantly prevented all of these alterations. These results suggest that Klotho can attenuate ox-LDL-induced oxidative stress in HUVECs through upregulating oxidative scavengers (SOD and NO) viaactivating the PI3K/Akt/eNOS pathway and depressing LOX-1expression.

**Conclusions:**

These results suggest that Klotho has a potential therapeutic effect on attenuating endothelial dysfunction and ameliorating atherosclerosis.

**Electronic supplementary material:**

The online version of this article (doi:10.1186/s12944-017-0447-0) contains supplementary material, which is available to authorized users.

## Background

Atherosclerosis is a common arterial disorder caused by a buildup of plaque. With increasing age, fat and cholesterol gather in the arteries to form the plaque. The accumulation of plaque then narrows the artery and impedes blood delivery to the body, which directly causes shortages in oxygen and nutrientsin various tissues. Plaque rupture causes blood clots that block coronary arteries resulting in a heart attack and obstruction of cerebrovascular vessels (stroke) [1]. Increased low density lipoprotein (LDL) and its oxidative form, oxidized low density lipoprotein (ox-LDL), inthe vascular sub-endothelium is characteristic of atherogenesis. These molecules attract and activate inflammatory cells, such as monocytes, T cells and macrophages. The activated macrophages thereupon release pro-inflammatory cytokines, reactive oxygen species (ROS), and proteolytic enzymes resulting in matrix degradation and atherosclerotic plaque destabilization [2]. Ox-LDL activity is mediated through scavenger receptors (SRs), including SR-A, SR-BI, CD36, and lectin-like oxidized low-density lipoprotein receptor-1 (LOX-1) [3]. It is reported that atherosclerosis-related factors [including tumor necrosis factor alpha (TNF$\alpha$), interleukin-1 (IL-1), interferon gamma (IFN$\gamma$), angiotensin II, endothelin-1, ox-LDL, free radicals, and fluid shear stress] up-regulate LOX-1 expression in vitro [2]. Studies using LOX-1 knockout [4] or overexpressing [5] mice suggested that LOX-1 is involved in the inflammatory response and lipid deposition in heart vessels. Moreover, the early stage of atherosclerosis is associated with increased levels of ox-LDL, oxidative stress, adhesion molecules and inflammatory cytokines in the vascular endothelium [6–8].

Klotho, which is a kind of anti-aging protein [9], is involved in various pathologies, such as atherosclerosis, heart damage, hypertension, acute kidney injury, chronic kidney disease, diabetes mellitus, and even cancer [7, 8, 10, 11]. In a Klotho-deficient mouse aging-model study, Kuro-o et al. reported that Klotho deficiency is closely related to cardiovascular diseases, such thatit enhances arteriosclerosis, extensive medial calcification of the aorta, and medial calcification and intimal thickening of medium-sized muscular arteries [9]. Moreover, some studies have revealed that Klotho may work as an important humoral factor involved in oxidative stress regulation, endothelial dysfunction, cell proliferation, and apoptosis [12–14]. Interestingly, similar to the up-regulation of ox-LDL, decrease inserum Klotho is also reported as a predictor of atherosclerosis [15]. Keles et al. recently revealed that Klotho may gain a protective ability against atherosclerosis and endothelial dysfunction in type 1 diabetes mellitus [15]. In addition, Navarro-Gonzále1z et al. analyzed the alteration of serum Klotho in 441 human coronary artery disease patients and concluded thathigh levels of soluble Klotho are correlated witha reduction incardiovascular risk [16]. Moreover, Klotho mitigates the effects of phosphate and FGF23 on contractility via increased NO production [17].

Base on the above findings, we explored the relationship between Klotho and atherosclerosis in an ox-LDL-induced endothelial cell injury model. We measured the protective effect of Klotho on ROS production, superoxide dismutase (SOD) activity, expression of LOX-1, and bioactive nitric oxide (NO) production in human umbilical vein endothelial cells (HUVECs).

## Methods

### Cell culture

HUVECs were kindly provided by Dr. Zhou (Department of Pathophysiology, Jinzhou Medical University, Jinzhou, Liaoning, China). The cells were cultured with Dulbecco’s modified Eagle medium (DMEM)-F12 (Gibco; EI Paso, Texas, USA) supplemented with 0.1 mg/ml heparin (Sigma-Aldrich; St. Louis, MO, USA), 0.05 mg/ml endothelial cell growth supplement (ECGS) (R&D Systems, USA), 10% fetal bovine serum (FBS) (Gibco) and Antibiotic Antimycotic Solution (Sigma-Aldrich) at 37 °C and 5% CO_2_. After pre-treatment with 200 pM Klotho protein (R&D Systems) for 1 h, sub-confluent HUVECs were stimulated with 50 μg/ml ox-LDL (Shanghai Jingke Chemical Technology Co., LTD, Shanghai, China) for 24 h. The cells were then used for ROS and SOD activity assays, and the cell lysates were used for Western blot analysis. The supernatant medium was collected for NO concentration detection.

### Cellular viability assay

Cellular viability was measured by the MTT uptake method. Briefly, HUVECs were plated at a density of 6.0 × 10^3^ cells/well in a 96-well plate. To analyze the effect of ox-LDL on HUVECs, the cells were treated with different concentrations (25, 50, 100, and 200 μg/ml) of ox-LDL for 24 h. In another experiment, HUVECs were pre-incubated with 100, 200, 400, and 800 pM of recombinant human Klotho (R&D Systems) protein for 1 h and subsequently treated with 50 μg/mlox-LDL for 24 h. These cells were then stained with 20 μl of 5 mg/ml MTT (Sigma-Aldrich) per well and incubated for 4 h at 37 °C. Continuously, the intracellular MTT purple formazan was solubilized with 150 μl of DMSO (Life, USA). The absorbance was detected at optical density (OD) 490 nm with auniversal microplate reader (ELx800, BioTeK, USA). Each experiment was performed three times.

### Measurement of intracellular malondialdehyde (MDA)

MDA levels were analyzed by commercially available colorimetric assay kits (Jian Cheng Bioengineering Institute, Nanjing, China) according to the manufacturer’s instructions. Briefly, pre-treated cells were harvested by scraping and then homogenized in RIPA buffer on ice. Cell lysates were then centrifuged at 12,000 g for 10 min at 4 °C to collect the supernatant. MDA levels were detected using a microplate reader at 532 nm. We used the bicinchoninic acid disodium (BCA) protein assay kit (Beyotime Biotech, Haimen, China) to quantify protein concentration. MDA content was expressed as nmol/mg protein.

### Determination of intracellular ROS production

ROS production was analyzed by staining with 2’ , 7’–dichlorofluorescindiacetate (DCFDA), a ROS-sensitive fluorescent dye (S0033, Beyotime Institute of Biotechnology, Nanjing, China) [18]. In brief, generation of intracellular ROS was detected using the fluorescent probe, DCFDA. Samples without DCFDA were used as negative controls. The positive control group was incubated with DCFDA and the Rousp (the positive control compound mixture, 50 mg/ml, which increased the level of ROS after ~20–30 min). HUVECs were inoculated in 6 cm culture dishes (25 × 10^4^/ml, 3 ml) for 24 h in the presence or absence of 200 pM Klotho for 1 h, followed by incubation withox-LDL (50 μg/ml) or solute for 24 h. Then the cells were stained with 10 μM/L DCFDA. After washing twice with cold phosphate buffered saline (PBS), the average fluorescence intensity of ROS in the cells was measured by flow cytometry (BD FACS Calibur TM, USA). Experiments were performed in triplicate.

### Measurement of SOD activity

Intracellular SOD activity was measured by the Total Superoxide Dismutase (T-SOD) assay kit (Nanjing Jiancheng Bioengineering Institute, Nanjing, China) based on the auto-oxidation of hydroxylamine. Targeted cells were lysed inPBS by ultrasonic pyrolysis (5 s sonication plus 5 s rest; 10 times). The homogenate was used for total SOD activity determination using thehydroxylamine method. The OD of the volume was obtained at 550 nm and each test was repeated over three times.

### Western blot analysis

Protein expression of gp91 phox, Cu/Zn-SOD, and LOX-1 in HUVECs was determined by western blot analysis. Briefly, HUVECs were homogenized and centrifuged to extract proteins. Protein concentrations were determined by BCA Protein Assay Kit (P0011, Beyotime Institute of Biotechnology, Nanjing, China) with a microplate reader (SH-1000, Corona Electric Co., Ltd, Japan) at OD 562 nm. Every 30 μg aliquot of protein was separated by a SDS-PAGE gel and transferred onto a nitrocellulose membrane. The transferred membrane was incubated overnight with specific polyclonal antibodies: anti-gp91 phox (1:500, Abcam, USA), anti-Cu/Zn-SOD (1:500, Novus Biologicals, USA), anti-LOX-1 (1:500, Abcam) and anti-β-actin (1:1000, Abcam). After washing three times, the blots were incubated with a corresponding secondary antibody and the probed protein was visualized by luminolchemiluminescenceBeyo ECL Plus (P0018, Beyotime Institute of Biotechnology, Nanjing, China) and finally detected by autoradiography exposure. The membrane was then re-blotted with β-actin antibody as a loading reference. Densitometry of the probed protein bands was analyzed for the aliquotsand normalized to corresponding β-actin, which was expressed as fold increases compared to the normal control group.

### Real-time quantitative PCR

We used quantitative PCR to quantify mRNA expression of Akt, phosphatidyl inositol-3 kinase (PI3K), inducible NO synthase (iNOS) and endothelial NOS (eNOS) in HUVECs after treatment. Total RNA was extracted from cells using TRIzol (Invitrogen, Carlsbad, CA, USA) and reverse transcribed according to the manufacturer’s instructions. Real-time PCR was performed using PowerUp™ SYBR® Green Master Mix (Thermo Fisher Scientific, Carlsbad, CA, USA) and con-ducted with the QuantStudio® 3 (Thermo Fisher Scientific). Primer sequences for real-time PCR are listed in Table 1. Glyceraldehyde-3-phosphate dehydrogenase (GAPDH) mRNA was used as anendogenous control.

**Table 1:** Sequences of RT-PCR primers used in this study

Gene name	Sequences
eNOS	Forward: 5’-GGA GAG GCT GCA TGA CAT TG-3’
	Reverse: 5’ -GGT AGA GCC ATA GTG GAA TGA C-3’
iNOS	Forward: 5’-AGA GAG ATC GGG TTC ACA-3’
	Reverse: 5’-CAC AGA ACT GAG GGT ACA-3’
GAPDH	Forward: 5’-CGG AGT CAA CGG ATT TGG TC-3’
	Reverse: 5’-AGC CTT CTC CAT GGT CGT GA-3’
Akt	Forward: 5’- TTG CTT TCA GGG CTG CTC A -3’
	Reverse: 5’- TCT TGG TCA GGT GGT GTG ATG -3’
PI3K	Forward: 5’- CGG TGA CTG TGT GGG ACT TA -3’
	Reverse: 5’- ACT GAT GTA GTG TGT GGC TGT -3’

### Measurement of NO production

NO concentration was measured by thenitrate reductase methodusing the Nitric Oxide assay kit (Nanjing Jiancheng Bioengineering Institute, Nanjing, China). In brief, the cellular supernatant culture medium treated with Klotho or ox-LDL was mixed with nitrate reductase. The OD volume was obtained at 550 nm by a spectrometer and each test was repeated over three times.

### Statistical analysis

All data are expressed as means ± SD. Comparisons of data among groups were performed by one-way ANOVA, and differences between two groups were analyzed by Student–Newman–Keuls test using SPSS 17.0 software. A *p*-value of less than 0.05 was considered significant for the differences.

## Results

### Klotho prevented the cytotoxic activity of ox-LDL in HUVECs

**Fig. 1:**
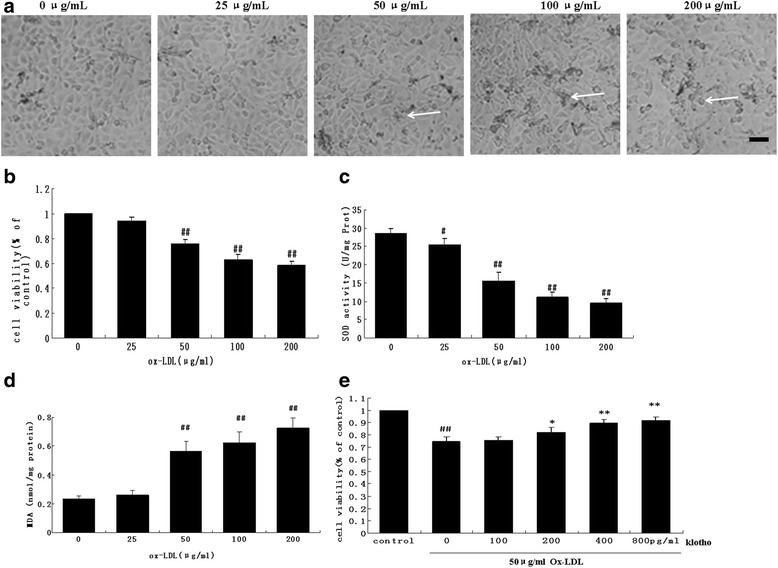
Effect of Klotho on cellular viability in HUVECs. Cells were exposed to various concentrations of ox-LDL (25, 50, 100, and 200 μg/ml) for 24 h. **a** HUVEC morphology was observed under an inverted phase contrast microscope (×10) following 24 h of ox-LDL treatment. Typical cellular fragmentations, vacuoles, and debris are arrowed. (Bar = 50 μm) (**b**) Cellular viability was detected by MTT assay. **c** and **d** SOD enzymatic activity and MDA levels in HUVECs were analyzed using commercially available assay kits. **e** HUVECs were pretreated with 100, 200, 400, and 800 pM of Klotho for 1 h, and then incubated with ox-LDL (50 μg/ml) for 24 h. Cellular viability was detected by MTT assay. Data are shown as mean ± S.D. (*n* = 3). Statistical differences are expressed as ^##^ *p* < 0.01 vs. control, *p < 0.05, **p < 0.01 vs. ox-LDL

As described above, ox-LDL may injurethe vascular endothelium through multiple pathways. We observed cellular morphology (Fig. 1a) and measured viability by MTT assay (Fig. 1b) in HUVECs after treatment with different concentrations (25–200 μg/ml) of ox-LDL to reveal the activity of ox-LDL under an optical microscope (Fig. 1a), we found that HUVECs were polygon or displayed short fusiform and were arranged as pebbles with clear borders between cells in the control group. In response to 25 μg/ml ox-LDLsome HUVECs presented with altered fragmentations. In response to 50 μg/ml ox-LDL vacuoles and fragmentations were found in some cells. When treated with 100 μg/ml, cell debris increased significantly. Following 200 μg/ml ox-LDL there was abnormal cellular morphology, more vacuoles, and fragmentations in some cells and the cellular arrangement was sparse. Ox-LDL concentrations greater than 50 μg/ml significantly decreased viability of HUVECs (*p* < 0.05) (Fig. 1b). We simultaneously measured oxidative stressby detecting SOD activity and MDA levels with various ox-LDL concentrations in HUVECs. As shown in Fig. 1c and d, 50–200 μg/ml ox-LDL significantly increased SOD activity and MDA levels compared to the untreated group (*p* < 0.01). No significant difference was found among the three groups (*p* > 0.05). Moreover, cell viability levels of ox-LDL-treated HUVECs were 0.98, 0.74, 0.43 and 0.28 at 12,24, 48 and 72 h, respectively (Additional file 1: Figure S1), which indicated that oxidative damage happened after 24 h of ox-LDL treatment. Based on these results, we used 50 μg/ml ox-LD for 24 h in the subsequent experiments.

To examine whether Klotho could affect this process, we pre-incubated the cells with different concentrations of the recombinant human Klotho protein (100 – 800 pM) for 1 h. Interestingly, >200 pM of recombinant human Klotho protein successfully prevented the decreased viability of HUVECs induced by 50 μg/ml ox-LDL (Fig. 1e) (*p* < 0.05). Importantly, a higher concentration of Klotho presented the most obvious ability to improve cell viability in ox-LDL-treated HUVECs. This data indicates that Klotho preventsox-LDL cytotoxicity in HUVECs.

### Klotho averted ROS production induced by ox-LDL in HUVECs

**Fig. 2:**
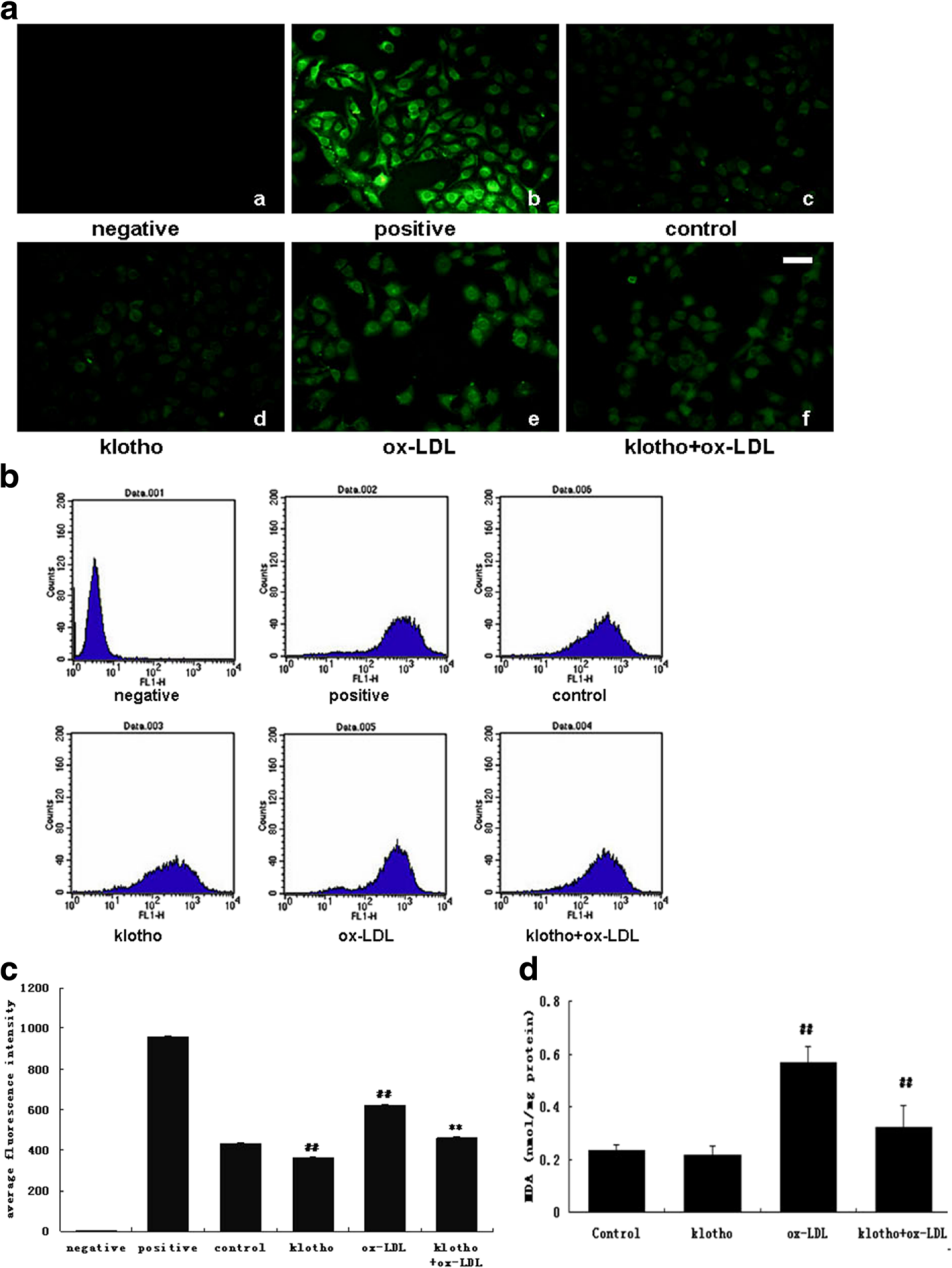
Klotho inhibited ROS production induced by ox-LDL in HUVECs. HUVECs were pre-incubated with 200 pM of recombinant human Klotho for 1 h, then treated with ox-LDL (50 μg/mL) for another 24 h. Ox-LDL alone and Klotho alone were used as controls. **a** Images observed under an inverted fluorescence microscope. (Bar = 50 μm) (**b**) Output figure of the fluorescence intensity detected by flow cytometry. **c** Average fluorescence intensity: Mean = total area under the peak/the total number of cells. Data are shown as mean ± S.D. **d** Lipid peroxidation was assessed by measuring the MDA levels in HUVECs treated with ox-LDL and/or Klotho (*n* = 3). Statistical differences are expressed as ^##^ *p* < 0.01 vs. blank control; **p < 0.01 vs. ox-LDL

Ox-LDL-activated macrophages can release ROS, which damage the vascular endothelium and promote pathogenic processes associated with atherosclerosis. Here, we initially investigated ROS production by staining with DCFDA (green) under different conditions in HUVECs (Fig. 2a). Under an inverted fluorescence microscope, the negative control was empty (black) (Fig. 2a *a*) and the positive control group was filled with green fluorescence (Fig. 2a *b*), which indicated successful acquisition of the fluorescence signal. Therefore, the average fluorescence intensity detected at the same batches of the same voltage condition was comparable. In the control group (Fig. 2a *c*), the fluorescence was very faint. In the sample analysis, ox-LDL clearly increased ROS green fluorescence (Fig. 2a *e*) compared to the un-treated negative control (Fig. 2a *c*). This increased ROS fluorescence induced by ox-LDL was attenuated by 200 pM Klotho (Fig. 2a *f*). The ROS signaling could be viewed with very slight stain in nu-treated (Fig. 2a *c*) and Klotho only treated samples (Fig. 2a *d*).

To quantify the ROS fluorescent stains, we continued detecting the average fluorescence intensity of the DCFDA-stained sample by flow cytometry (Fig. 2b). The results of the quantitative analysis were then compared in a histogram (Fig. 2c). The average fluorescence intensity of the negative control group was 3.13 ± 0.19; the positive control group was 959.16 ± 5.55. Compared with the blank control group (430.34 ± 6.76), the average fluorescence intensity of the ox-LDL group (621.79 ± 7.06) was significantly enhanced (*p* < 0.01), indicatingthat ox-LDL treatment increased ROS production in HUVECs. However, pre-treating with Klotho significantly prevented the ox-LDL-induced ROS stain (458.74 ± 4.64) compared to the ox-LDL group (*p* < 0.01). Analogously, Klotho significantly reversed lipid peroxidation compared to the ox-LDL group (*p* < 0.01) (Fig. 2d).

### Klothoincreased total SOD activity and up-regulated Cu/Zn-SOD expression in HUVECs

**Fig. 3:**
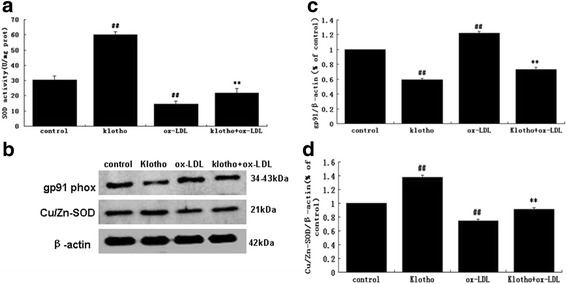
SOD, Cu/Zn-SODandgp91 phox in ox-LDL and Klotho-treated HUVECs. Cellular treatment was the same as described in Fig. 2. **a** Intracellular SOD activity was determined by the hydroxylamine method. **b**, **c** and **d** Western blotanalysis of Cu/Zn-SOD and gp91 phoxexpression in pre-treated HUVECs. Densitometry of the probed bands from the western blot were analyzed by Image J2x. Values are means ± S.D. (*n* = 3). Statistical differences are expressed as ^##^ *p* < 0.01 vs. control; **p < 0.01 vs. ox-LDL

SOD and its metal cofactor type Cu/Zn-SOD are important free radical scavengers that work through multiple mechanisms to prevent damage from reactive (O_2_^−^) [19]. To investigate whether SOD and/or Cu/Zn-SOD participate in the anti-oxidative mechanism of Klotho, we explored the effects of Klotho on the intracellular SOD/Cu/Zn-SOD activities in HUVECs. SOD activity was measured using theT-SOD assay kit. Compared to the controls, Klotho alone significantly increased intracellular SOD enzyme activity (*p* < 0.01); in contrast, ox-LDL alone significantly decreased intracellular SOD enzyme activity (*p* < 0.01). However, Klotho notably attenuated the reduction of the SOD activity induced by ox-LDL (*p* < 0.01) (Fig. 3a). These results show that Klotho can promote SOD activity in normal cells and diminish the decline of SOD activity caused by ox-LDL.

These results were then confirmed at the protein level by western blot analysis. We measured expression of Cu/Zn-SOD, a kind of cytoplasm SOD, and gp91 phox[catalytic subunit of nicotinamide adenine dinucleotide phosphate (NADPH) oxidase]. As shown in Fig. 3b-d, Klotho inhibited gp91 phox expression, but up-regulated expression of Cu/Zn-SOD (*p* < 0.01). Ox-LDL decreased expression of Cu/Zn-SOD, but enhanced gp91 phox compared to the control (*p* < 0.01). Nevertheless, Klothoattenuated these alterations of both gp91 phox and Cu/Zn-SOD expression induced by ox-LDL compared to the ox-LDL group (*p* < 0.01). This data suggests that Klotho alone not only increases expression of Cu/Zn-SOD and decreases expression of gp91 phox, but also reverses the decreased expression of Cu/Zn-SOD and the increased expression of gp91 phox induced by ox-LDL in HUVECs.

### Klotho enhanced NO productionand Akt/eNOS expressionin HUVECs

**Fig. 4:**
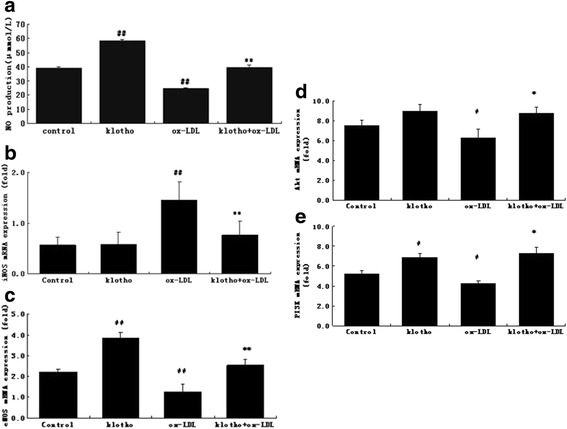
Klotho regulated NO production in HUVECs. **a** NO production in pre-treated HUVECs. **b**, **c**, **d**, and **e** mRNA levels of iNOS, eNOS, PI3K, and Akt were measured by real time PCR. Values are means ± S.D. (*n* = 3). Statistical differences are expressed as ^#^ *p* < 0.05*vs*. control; *p < 0.05 *vs*. ox-LDL. ^##^ *p* < 0.01 vs. control; **p < 0.01 vs. ox-LDL

NO, a free radical and vasodilative factor, is a signaling molecule in the cardiovascular system. Therefore, NO production was investigated in the HUVEC cellular culture medium (Fig. 4a). Compared with the controls, NO production was significantly decreased by ox-LDL (*p* < 0.01). Klotho not only significantly enhanced NO production (*p* < 0.01) but also rescued the decreased NO level induced by ox-LDL (*p* < 0.01).NO synthesis is regulated by nitricoxide synthase including both eNOS and iNOS [20] As shown in Fig. 4b and c, Klotho significantly inhibited iNOS expression, but up-regulated eNOS expression compared to the ox-LDL group (*p* < 0.01). These results indicate that Klotho can increase NO production in normal cells and attenuate the declined NO release caused by ox-LDL.

To investigate whether the increased eNOS was associated with activation of PI3K/Akt, we quantified the mRNA levels of PI3K and Akt. Compared with the controls, ox-LDL significantly decreased PI3K and Akt expression (*p* < 0.05). Klotho attenuated the ox-LDL mediated changes in PI3K and Akt expression compared to the ox-LDL group (*p* < 0.05) (Fig. 4d and e). This data suggests that Klotho may rescue the ox-LDL-induced inhibition of NO production via activation of Akt/eNOS pathway in HUVECs.

### Klotho reversed the ox-LDL-induced up-regulation of LOX-1 in HUVECs

**Fig. 5:**
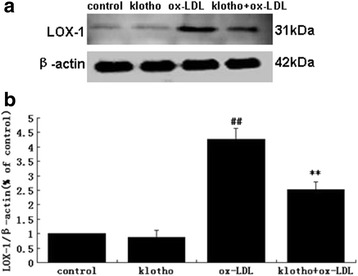
LOX-1 expression in HUVECs. Under similar treatment conditions as above, LOX-1 was detected by western blot (**a**), and densitometry (**b**) of the probed LOX-1 was quantified by Image J2x and normalized to β-actin bands. Values are means ± S.D. (*n* = 3). ^##^ *p* < 0.01 vs. control; **p < 0.01 vs. ox-LDL

LOX-1, anox-LDL receptor, is acrucial factor in atherosclerosis pathogenesis. Thus, we analyzed expression of LOX-1 in HUVECs treated with different conditions. In western blot analysis, ox-LDL considerably increased LOX-1expression (*p* < 0.01) compared to the control (Fig. 5a). However, this up-regulation of LOX-1 was obviously reversed by pretreatment with Klotho in contrast to theox-LDL group (*p* < 0.01). Moreover, in contrast to the control, Klotho alone did not influence LOX-1 expression (*p* > 0.05). This data indicates that Klotho may rescue ox-LDL-induced injury through the inhibition of LOX-1 in HUVECs.

## Discussion

Klotho is reported to be involved in maintaining cardiovascular health through its multiple anti-inflammatory and anti-oxidative effects [7], although the molecular mechanisms for these effects are not yet defined. In our study, we examined the protective effects of recombinant Klotho protein on endothelium cell (HUVECs) injury induced by ox-LDL. Concerning the underlying atherogenesis, the “oxidative modification hypothesis” indicates that LDL oxidation is the initial affair in atherosclerosis [21]. Alterations in LDL and ox-LDL are believed to occur very early, even before the apparent symptoms and diagnosis of atherosclerosis [22, 23]. Chrysohoou et al. [24] found a relation between pre-hypertension and declined serum antioxidant capacity/enhanced ox-LDL level, which hints at initial pathological changes in a large sample volume of cardiovascular disease-free people. Here, our data clearly showed a cytotoxic activity of ox-LDL on vascular endotheliumat 50 μg/ml in vitro (Fig. 1b). The accumulation of ox-LDL in the vascular wall results in vascular dysfunction early in the development of atherosclerosis. Recombinant Klotho at 200 pM prevented this damage induced by ox-LDL in HUVECs (Fig. 1e). This result revealed the ability of Klotho to maintain cardiovascular health in our conditions.

ROS are reactive oxygen-containing chemical species including peroxides, superoxide, hydroxyl radical, and singlet oxygen. ROS, in a biological condition, have essential activity in cell signaling and homeostasis. However, in environmental disorders (e.g., oxidative stress), ROS production is increased dramatically, which may cause substantial injures to cell structures [25]. Previous research showed that ROS and ox-LDL production are increased throughout atherosclerosis progression [26]. ROS overproduction has been implicated in endothelial injury and extracellular/intracellular oxidative stress [27, 28]. In our study, ox-LDL elicited a cytotoxic effect that directly up-regulated ROS production in HUVECs (Fig. 2a). Cominacini et al. proved that ox-LDL improves ROS formation in HUVEC through association with a specific endothelial receptor, which may trigger nuclear factor-κB (NF-κB) activation to induce ROS formation [29]. In addition, antioxidant compounds may improve vascular health and reduce atherogenesis by inhibiting ROS production and oxidative modification of LDL [30–32]. Therefore, we investigated whether Klotho can inhibit ROS formation induced by ox-LDL. Our results showed that Klotho alone not only reduced ROS levels in normal HUVECs but also prevented ox-LDL-induced ROS (Fig. 2). This finding was reported previously by Rakugi et al. [14] who showed that Klotho prevented AngiontensinII-induced ROS over production. Moreover, Yang et al. indicated that Klotho could prevent uremic toxin indoxyl sulfate-induced endothelial cell dysfunction via reducing ROS production and NF-κBactivation [33]. Our findings support these studies. Based on these data, we speculate that Klotho protects against ox-LDL-induced endothelial dysfunction partly through suppressing ROS over production, thus inhibiting the initiation and progression of atherosclerosis.

Oxidative stress is usually caused by either an enhanced oxidant and/or a lessened antioxidant system. Based on this, we continuously analyzed the effects of Klotho on ROS synthases and ROS scavenging enzymes. SOD are a group of metalloenzymes (containingCu and Zn), that catalyze disproportionate superoxide free radicals into either ordinary molecular oxygen (O_2_) or hydrogen peroxide (H_2_O_2_) [34]. NADPH-oxidase is a major oxidase producing ROS. And gp91 phox is a subunit of the NADPH-oxidase [35]. In our study, ox-LDL significantly depressed both total SOD activity and Cu/Zn-SOD expression and up-regulated gp91 phox expression in HUVECs (Fig. 3). Notably, Klotho successfully attenuated the ox-LDL-decreased total SOD activity and Cu/Zn-SOD expression, as well as the ox-LDL-enhanced gp91 phox expression in HUVECs (Fig. 3). Klotho was reported to enhance Mn-SOD expression in HUVECs and Klotho gene transfer decreased Nox2 protein expression, but did not affect Nox2 mRNA expression in rat aorta smooth muscle (RASM) cells [14, 36]. Furthermore, Klotho gene transfer reduced intracellular superoxide production and oxidative stress in RASM cells [36]. Klotho prevented ROS increases and cellular apoptosis induced by TNFα [36]. Recent reports also revealed that inhibition of vascular oxidative stress may prevent cardiovascular diseases (CVD) [37–39]. Therefore, we inferred that Klotho protects the vascular endothelium through attenuating oxLDL-induced oxidative stress and prevents the development of atherosclerosis. The signaling pathway of the Klotho-induced suppression of Nox2 expression may be mediated by the cyclic AMP (cAMP)- protein kinase A (PKA) pathway [14, 40]. However, the detailed mechanisms of the modulation of gp91 phox and Cu/Zn-SOD by Klotho require further exploration.

It is widely known that dysfunction of the endothelial nitric oxide synthase (eNOS) (especially endothelial NOS) activity leads to the reduction of NO bioavailability, which contributes to atherosclerosis. We demonstrated that Klotho not only increased NO production and eNOS in normal cells, but also reversed the declined NO and eNOS caused by ox-LDL (Fig. 4). Similarly, Yoshihiro et al. showed that Klotho recovers eNOS phosphorylation reduced by TNFα in HUVECs [39]. Our data are consistent with the earlier report, which illustrated that Klotho amplified NO production in endothelial cells [14]. PI3K, which is a heterodimeric enzyme, plays animportant role in proliferation and apoptosis, while its downstream serine-threonine kinase, Akt, transmits survival signals from growth factors [41]. The PI3K/Akt pathway is a very important pathway that is involved in ox-LDL-induced HUVEC proliferation and apoptosis [42]. It was reported that Aktactivatese NOS to induce NO production [43]. The current study suggests that Klotho may rescue ox-LDL-induced inhibition of NO production through the activation of Akt/eNOS pathway in HUVECs. Similarly, some natural compounds also protect endothelial cells from ox-LDL-induced apoptosis by modulating the PI3K/Akt/eNOS pathway [44]. In contrast, many studies indicated that decreased NO is another key mechanism underlying endothelial dysfunction, which is interrelated with decreased expression of the Klotho gene [33, 45–47]. In addition, it has been proven that Klotho protects the cardiovascular system through endothelium-derived NO production by humoral pathways [47]. Thus, Klotho protects against endothelial dysfunction.

LOX-1 is a major ox-LDL receptor in endothelial cells. Ox-LDL mostly signals through LOX-1. Studies with LOX-1 knockout or LOX-1 over expressing mice indicated a key contribution of LOX-1 in the inflammatory response and lipid deposition in blood vessels [4, 5]. Xu et al. found that administration ofanti-LOX-1 antibodies prevented atherosclerosis by decreasing cellular events, including endothelial dysfunction, monocyte adhesion, proliferation, migration, and apoptosis of smooth muscle cells, foam cell formation, platelet activation, and plaque instability [48]. Therefore, obstruction of the LOX-1 receptor results in inhibition of ox-LDL activity. Based on this point, we analyzed the effectof Klotho on LOX-1. Interestingly, ox-LDL tremendously up-regulated LOX-1 expression in HUVECs, and recombinant Klotho protein effectively decreasedthis up-regulation (Fig. 4). This result suggests that LOX-1 pathway inhibition is one of the mechanisms by which Klotho maintains blood vessel heath.

## Conclusions

The anti-oxidative activity of Klotho may be mediated through upregulation of oxidative scavengers (SOD and NO) after activation ofthe PI3K/Akt/eNOS pathway and downregulation of LOX-1 expression. These findings indicate that the anti-oxidative action of Klotho plays an important role inprotecting endothelial cells. This study provides the experimental evidence supporting the clinical application of Klotho to prevent or treat atherosclerosis.

## Additional file

Additional file 1: Figure S1. 50 µg/mL ox-LDL treatment induced decrease of cell viability in time dependent manner in HUVECs. Cells were treated with 50 µg/mL ox-LDL for indicated time. Cell viability were assayed by MTT assays. The results were presented as mean±SD from three independent experiments. ^##^ *p*< 0.01. (JPG 8 kb)

## Abbreviations

CVD: Cardiovascular diseases
eNOS: Endothelial nitric oxide synthase
HUVECs: Human umbilical vein endothelial cells
iNOS: Inducible nitricoxide synthase
LOX-1: Lectin-like ox-LDL receptor 1
NO: Nitric oxide
ox-LDL: Oxidized low density lipoprotein
ROS: Reactive oxygen species
SOD: Superoxide dismutase
