# Supplementary figures and images for: Klotho ameliorates oxidized low density lipoprotein (ox-LDL)-induced oxidative stress via regulating LOX-1 and PI3K/Akt/eNOS pathways
Source: Lipids Health Dis. 2017 Apr 13;16:77. doi: 10.1186/s12944-017-0447-0 (PMC5390438; doi:10.1186/s12944-017-0447-0)

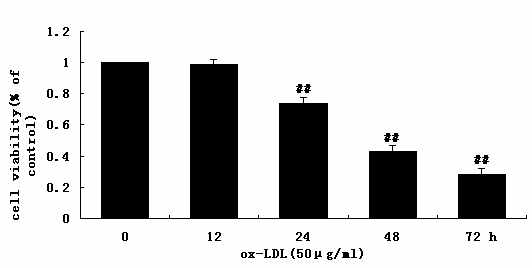

Supplement: Additional file 1: Figure S1. — 50 µg/mL ox-LDL treatment induced decrease of cell viability in time dependent manner in HUVECs. Cells were treated with 50 µg/mL ox-LDL for indicated time. Cell viability were assayed by MTT assays. The results were presented as mean±SD from three independent experiments. ## p< 0.01. (JPG 8 kb) [file 12944_2017_447_MOESM1_ESM.jpg]
